# miREE: miRNA recognition elements ensemble

**DOI:** 10.1186/1471-2105-12-454

**Published:** 2011-11-24

**Authors:** Paula H Reyes-Herrera, Elisa Ficarra, Andrea Acquaviva, Enrico Macii

**Affiliations:** 1 Department of Control and Computer Engineering, Politecnico di Torino, Corso Duca degli Abruzzi 24, 10129 TO, Italy

## Abstract

**Background:**

Computational methods for microRNA target prediction are a fundamental step to understand the miRNA role in gene regulation, a key process in molecular biology. In this paper we present miREE, a novel microRNA target prediction tool. miREE is an ensemble of two parts entailing complementary but integrated roles in the prediction. The Ab-Initio module leverages upon a genetic algorithmic approach to generate a set of candidate sites on the basis of their microRNA-mRNA duplex stability properties. Then, a Support Vector Machine (SVM) learning module evaluates the impact of microRNA recognition elements on the target gene. As a result the prediction takes into account information regarding both miRNA-target structural stability and accessibility.

**Results:**

The proposed method significantly improves the state-of-the-art prediction tools in terms of accuracy with a better balance between specificity and sensitivity, as demonstrated by the experiments conducted on several large datasets across different species. miREE achieves this result by tackling two of the main challenges of current prediction tools: (1) The reduced number of false positives for the Ab-Initio part thanks to the integration of a machine learning module (2) the specificity of the machine learning part, obtained through an innovative technique for rich and representative negative records generation. The validation was conducted on experimental datasets where the miRNA:mRNA interactions had been obtained through (1) direct validation where even the binding site is provided, or through (2) indirect validation, based on gene expression variations obtained from high-throughput experiments where the specific interaction is not validated in detail and consequently the specific binding site is not provided.

**Conclusions:**

The coupling of two parts: a sensitive Ab-Initio module and a selective machine learning part capable of recognizing the false positives, leads to an improved balance between sensitivity and specificity. miREE obtains a reasonable trade-off between filtering false positives and identifying targets. miREE tool is available online at http://didattica-online.polito.it/eda/miREE/

## Background

MicroRNAs (miRNAs) are short 19-23 nucleotide non-coding RNAs that have a central role as post-transcriptional repressors of gene expression acting on messenger RNA (mRNA). The microRNA binds miRNA recognition elements (MRE or binding sites) mostly in the mRNA untranslated region leading to translational repression or mRNA degradation [1,2]. The mechanisms that mediate gene regulation by the miRNA are still open to discussion, regardless the intensive research on the field. To date, computational prediction of miRNA targets represents a fundamental step in order to understand the microRNA operation and contribution toward cell functions [3,4].

MicroRNA target prediction is based on (1) several features related to structural characteristics of the miRNA-MRE duplex interaction and (2) common characteristics in the MRE vicinity representing the accessibility of the MRE for the miRNA. Among the features related to the interaction, the complementarity of miRNA and MRE in the so called *seed *region is considered a key one. The seed is a subsequence (1-8 nt) of miRNA in the 5' extreme, whose complementary sequence is commonly found in the experimentally supported MREs [5]. For this reason the seed is usually considered in almost all the target prediction tools currently available.

Besides the many similarities, computational approaches for the miRNA target prediction have been developed using various approaches. From this perspective, two main categories can be identified: Ab-Initio methods and *Machine Learning *(ML) approaches. Ab-Initio methods make use of experimental data available indirectly, extract informative features from them and use the features to feed a computational model. Popular tools belonging to this category are miRanda [6], Targetscan (TargetscanS) [7] and Pictar [8,9]. Machine learning approaches have been later introduced to face the high false positive rate problem of Ab-Initio methods [10-12]. They make direct use of the experimental data to learn the features or characteristics of the miRNA binding site, which are then used to make a similarity comparison with the MRE samples. As a result, the effectiveness of machine learning approaches is more directly dependent on the availability and quality of experimental data. In particular, their effectiveness is impacted by the lack of information regarding the true negatives. However, their advantage is that they can easily integrate information coming from new data to improve their selectivity. In order to overcome this pitfall, alternative tools such as miTarget [12], NBmiRTar [13], miRTif [11] and TargetMiner [14] have been developed exploiting different methods to expand the negative set. Recently, hybrid methods where a machine learning approach is preceded by an Ab-Initio part or a sequencing algorithm have been proposed [13-17]. The objective of the first part is mainly to locate regions of the target mRNA that show complementarity with the miRNA seed. The second part is then applied to improve the selection of these regions by looking at other features.

However, in current hybrid systems the two parts are designed independently, resulting in most of the cases from the composition of two independent tools [13,14]. As a consequence, these methods are not designed as an integrated tool. We believe that the joint optimization of the Ab-Initio and ML parts can lead to better results in terms of specificity and sensitivity of the tool.

In this paper we present a target prediction method composed of an Ab-Initio and a Machine Learning module, where the effectiveness of the innovative Ab-Initio method based on a genetic algorithm and its integration with the ML part lead to an enhanced accuracy with respect to state-of-the-art prediction tools. We exploited the integration of the two parts in two ways: i) The recognition of features is optimally split between the two parts; ii) The Ab-Initio part is used to generate negative samples for the ML part to improve its recognition capabilities.

In particular, the Ab-Initio method we developed first generates a set of candidate binding sites on the basis of miRNA site characteristics in a mRNA-target independent way through a genetic algorithm. This feature allows the decomposition of the problem of target recognition in two sub problems, namely (i) the individuation of miRNA recognition elements (performed by the Ab-Initio part) and (ii) the evaluation of the impact of target mRNA, such as the surroundings of the site, on the accessibility of a certain miRNA (performed by the ML part). This decomposition is not possible in methods where the Ab-Initio part is based on a direct target search of binding sites and subsequent filtering [13,14]. Furthermore, the Ab-Initio part and its generation of candidate binding sites is exploited for the creation of additional negative samples, with respect to the experimental ones. These negative samples have been used to train the Machine Learning part.

As a whole, the entire method is able to overcome the most relevant drawbacks of computational miRNA target prediction, namely the experimental data-dependency and notorious false positive rate. To discuss its effectiveness, we report the results we obtained by comparing miREE with state-of-the-art prediction tools that show a consistent improvement in the balance between specificity and sensitivity. We performed a detailed characterization and comparison of both Ab-Initio and ML parts to highlight their contribution. As part of the characterization, we provide an extensive discussion about the biological information exploited in the tool and we provide a ranking of the biological features used in the ML part based on their impact on the achieved accuracy. Performance tests have been carried out on experimental data provided by heterogeneous experimental validation methods, evidencing the robustness of miREE capability to detect miRNA recognition elements.

## Methods

### Data Set

**Figure 1:**
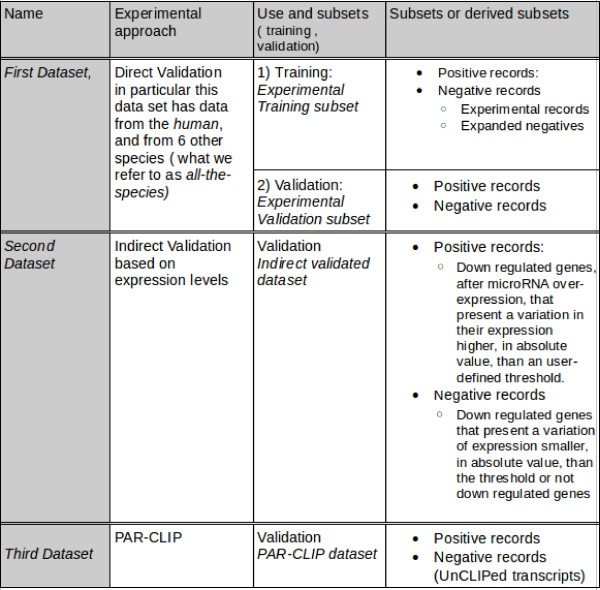
**Table with Datasets, subsets and experimental approach used to obtain them. Note that the dataset names are in Italic**.

To better explain the different datasets used for the evaluation of the method performance, we first briefly describe the currently available experimental data. The data regarding the miRNA:mRNA interactions has been obtained through (1) direct validation where the experimental methods allow to validate the specific interaction (even the MRE in detail can be provided), or through (2) indirect validation, based on gene expression variations obtained from high-throughput experiments where the specific interaction is not validated in detail and consequently the specific binding site is not provided. In this study we used three different datasets: the first dataset was extracted from public databases [18] and [19], the second dataset was extracted from a proteomic study [20], and the third dataset was extracted from a recent high-throughput CLIP study [21]. These 3 main datasets and their main subsets can be seen in Figure 1, nonetheless a more detailed description of the datasets can be found at follows.

To perform characterization and evaluation of miREE performance, we devised the first dataset by extracting those records obtained from direct experimental methods that contain information about binding sites. This dataset is suitable to validate those methods, such as miREE, based on structural recognition elements because is a dataset with a solid experimental support regarding the specific miRNA-mRNA interactions. In particular, this first dataset was extracted from miRecords Version 1 [18] and Tarbase Version 5 [19] databases with experimental data regarding miRNA-target interactions. The selection was needed because in both databases there are both binding sites with evidence (direct validation) and mRNAs where the miRNA regulation was experimentally confirmed based on gene and protein expression (indirect validation), for which there is not an experimental evidence for the specific site. In this last case the databases provide predictions obtained from Ab-Initio methods [6,8,7] to indicate possible interactions of the miRNA:mRNA pair. As such, the records from indirect validations were discarded. The selected sites corresponding to the selected records were located in the corresponding mRNA sequences and features regarding the site-miRNA interaction and the neighborhood characteristics were obtained. At the end there were 324 Positives (293 from miRecods, 31 from Tarbase) and 46 Negatives (40 from miRecods, 6 from Tarbase), for seven species.

**Figure 2:**
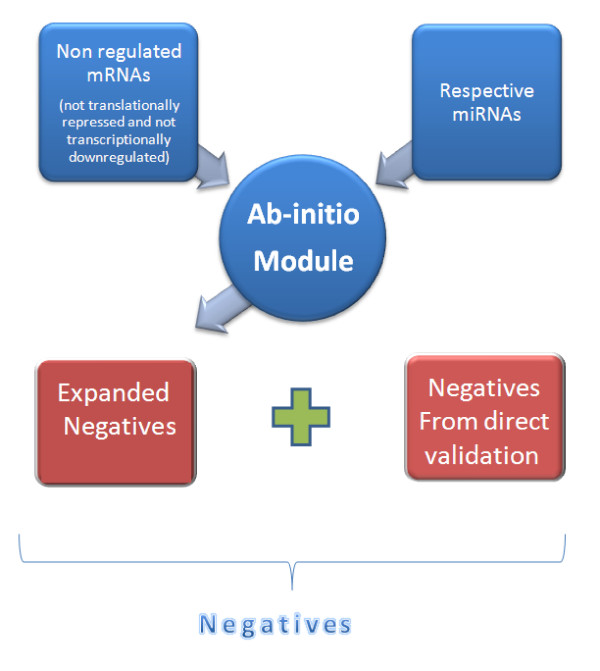
**Negative records expansion**. Block diagram for the expansion of the negative records using the Ab-Initio part of miREE.

Since the number of negative records was deficient with respect to the number of positive records, the former was expanded as follows. First of all we selected a set of genes that were non-regulated targets because they were not translationally repressed or transcriptionally downregulated by a specific miRNA, based on experimental data from indirect validations. Note that it was required for the genes to have a minimum presence in the cell types where the expression was measured. Consequently predictions for the selected non-regulated targets were considered as negative samples with evidence, because they are part of non-regulated genes for the identified miRNAs experimentally supported. Hence, any subsequence from those genes is a negative sample. Thus, we considered non-regulated targets with indirect validation and we made predictions on those non-regulated mRNA targets for the considered miRNAs using our Ab-Initio method (Section Method) and we integrated them with the Negatives derived from direct validation into the negative records as it can be seen in Figure 2, obtaining 305 samples for a set of 6 miRNA-Target couples from *Caenorhabditis elegans*, *Mus musculus*, *Homo sapiens*. The first dataset was finally made of a total of 675 records, 324 Positives and 351 Negatives. These records correspond to the experimental data for 7 species, 128 miRNAs and 183 mRNAs. The 7 species are *Caenorhabditis elegans*, *Mus musculus*, *Homo sapiens*, *Drosophila melanogaster*, *Rattus norvegicus*, *Danio rerio*, *Kaposi sarcoma-associated herpesvirus*.

We define the part of this dataset used for training as *Experimental Training Subset*, while the part used for testing as *Experimental Validation Subset*.

To perform a more complete evaluation of miREE, we used two additional datasets. The above mentioned second dataset is from [20], it contains targets determined by indirect validation, so we called it the *indirect validated *dataset. Even if it does not provide information about direct structural interaction between miRNA and mRNA, we considered this dataset mainly because it was used in [22] to compare the performance of 8 Ab-Initio methods. Thus, we exploited the results presented in [22] in order to compare miREE with these Ab-Initio methods. In addition the third dataset consists of a high throughput CLIP (Crosslinking Immunoprecipitation) data. In particular we considered the PAR-CLIP data [21] (exploiting the *Photoactivatable-Ribonucleoside-Enhanced Crosslinking and Immunoprecipitation *method) and selected a subset composed of 596 binding sites present in the 3'UTR for a set of 11 miRNAS, from the top 25 expressed miRNAs. In particular, the 11 miRNAs selected were not contained in our training set. From the CLIP repository, data from PAR-CLIP method was selected because it represents an improved method for the isolation of RNA bound to ribonucleic complexes (RNP) compared to the original CLIP method. In particular, we selected the PAR-CLIP method because it uses photoreactive nucleosides that enable to enhance the crosslinking facilitating the precise identification of RNPs binding sites. The PAR-CLIP protocol is interesting because it provides experimental evidence for mRNA sites where the miRNP complex (miRNA+RISC) bind, and the specific sites of interaction in the mRNAs are provided using an innovative type of experimental procedure which is based on direct validation, even if there is not detailed information about the specific associations of miRNA-mRNA target pairs.

### Method

**Figure 3:**
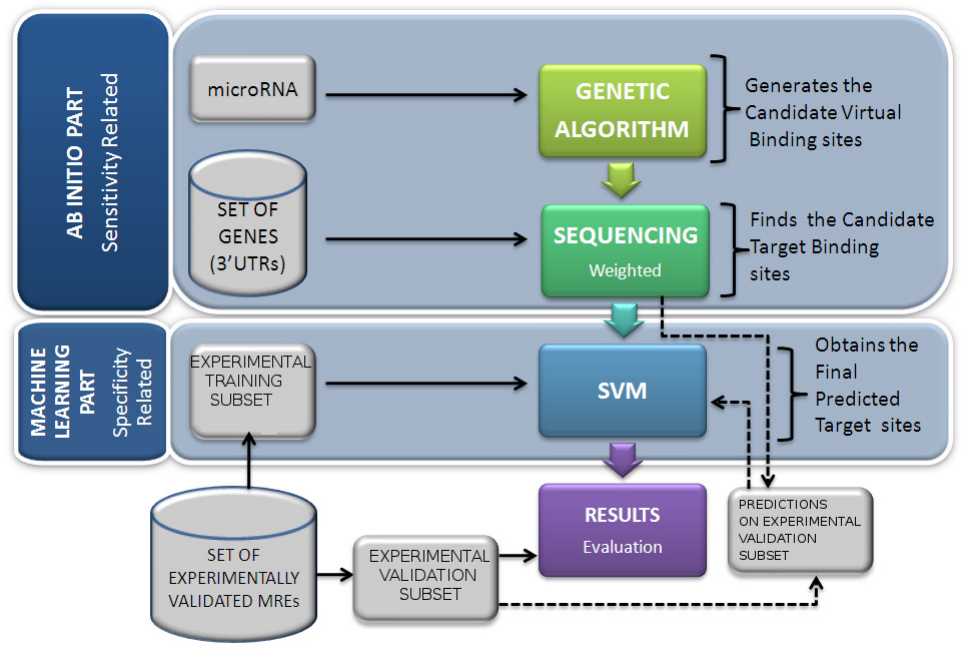
**General schema for the miRNA target prediction**. Block diagram of the proposed method miREE. Composed of the 2 parts Ab-Initio part and Machine learning part.

We propose an integrated method composed by two parts, the global scheme can be seen in Figure 3. The first part mainly impacts the sensitivity of the method, while the second part is more responsible for its specificity. In the first part, called Ab-Initio part, predictions for a miRNA are made using a Genetic Algorithm. The purpose of this part is to generate a population of sequences that capture the main structural characteristics studied in the miRNA recognition elements, as it will be deeply described later in this Section. The set of sequences generated is called *Candidate virtual binding sites*. Further, this population is mapped into the mRNAs UTRs, using a weighted sequencing, to find the *Candidate Target Binding Sites*. The second part, called Machine Learning part, consists in a SVM classifier that was previously trained using MRE positive and negative sites (experimentally validated) and is used to reduce the false positives. This SVM classifier takes as input the *Candidate Target Binding Sites *and outputs the *Final Predicted Targets Sites*.

#### Ab-Initio Part (Sensitivity module)

The Ab-Initio part is composed of two modules. The first module (*miRNA module*) concerns miRNA-MRE duplex specific characteristics, the second module (*mRNA module*) considers environmental characteristics for the binding site that have an influence on the accessibility of the miRNA to the site.

##### miRNA module

The task of this module is to generate a set of candidate binding sites (the most promising binding sites) for a specific miRNA. These sequences will be called *Virtual Sites *thereafter. The *Virtual sites *are generated using a Genetic Algorithm (GA), where the individuals are sequences of nucleotides that represent possible binding sites. The GA starts from a random population of 10, generates at each generation 100 sequences using the mutation and crossover operators, and selects the fathers of the next generation using the truncation selection method [23]. To generate the next generation the mutation occurs with a uniform probability of 0.2 and the crossover operator is fixed at a single-point at the nucleotide in the position N-8 where N is equal to the length of the miRNA. The point is selected to promote the seed complementary site in most of the Virtual sites. Thus the GA starts from a random population that evolves coherently to the fitness function. The algorithm stops when the results of the fitness function do not improve for a couple of generations. At each generation, the sequence with the best value computed by the fitness function is taken for a candidate set. The final *Candidate virtual binding sites *set is composed of the sequences from the candidate set that had an associated fitness function value better than the fitness function value obtained with the complementary-seed sequence of the respective miRNA. Therefore the genetic algorithm obtains a variety of sequences representing promising candidate binding sites for a given miRNA.

It is worth noting that the generation of virtual sequences, that represent sequences with characteristics similar to the miRNA responsive elements, allows a virtual sequence with gaps and bulges. On the other side, a remarkable value of the associated fitness function is necessary in order to enable the virtual sequence to belong to the Candidate virtual binding sites Set. This means that the virtual sequence, including gaps and bulges, still possesses characteristics that favor the binding with the miRNA, for example a well-binding in the region towards the 3'end of the microRNA.

The idea behind the design of the Ab-Initio part is to have a target-independent generation of candidates. The target independency intuitively provides means to identify unknown targets. By using the GA, virtual binding sites are generated based only on the characteristics of the microRNA under study. After this first target-independent step, virtual sequences are mapped into the targets by using the sequencing search to find real subsequences from the mRNA sequences very close to the virtual binding sites. This is conceptually different from directly looking into targets to find out subsequences with suitable binding characteristics. It must be noted that this approach, that is the generation of target independent virtual binding sites can be useful for comparing the characteristics of different microRNAs and to evaluate the impact of the same microRNA across different species, by reusing the same set of virtual candidates. Finally, the fitness function lends itself to be refined once additional features are determined from experiments.

The fitness function was previously set using characteristics that intend to capture the nature of miRNA responsive elements. We characterized the behavior for three different fitness functions: *fitness*_0_, *fitness*_1 _and *fitness*_2_. These functions represent different combinations of parameters related to the duplex (miRNA-mRNA) interaction and in particular specific regions: the seed, the region outside the seed (out-seed) and the section from the 13th to the 16th nucleotide (13-16). Note that the combination of several features in the fitness function allows obtaining sites where either the features are well defined or the feature compensation is present.

The mentioned characteristics are the duplex hybridization energy *Energy*_*Duplex*_, *Energy*_*seed*_, *Energy*_*out-seed*_, *Energy*_13-16_, and the number of bases unpaired (BU) in the respective regions. In order to weight the contribution of the seed region with respect to the non-seed region the parameter *α *was introduced in *fitness*_1 _and *fitness*_2_. These functions are described in Table 1.

**Table 1:** Fitness functions

***fitness***_**0**_	***Energy***_***seed ***_**+ *Energy***_**13**__**-**__**16**_
*fitness*_1_	$\frac{Energy_{Duplex}}{\alpha BU_{seed}+BU_{out-seed}+1}$

*fitness*_2_	$\alpha \frac{Energy_{seed}}{BU_{seed}+1}+\frac{Energy_{out-seed}}{BU_{out-seed}+1}$

It can be observed that *fitness*_0 _enhances those elements having specific regions (i.e. seed and miRNA 13-16nt) characterized by a notable contribution to the hybridization energy. Instead *fitness*_1 _promotes higher stability through the duplex hybridization energy and a small number of bases unpaired in the seed and out-seed regions. Finally, *fitness*_2 _promotes stability in the seed and out-seed regions through the respective hybridization energies, and a small number of bases unpaired in the respective regions. In *fitness*_1 _and *fitness*_2 _the contribution of the seed region was weighted using the parameter *α*. The impact of this parameter has been explored as discussed in Section Fitness parameters. Each one of these functions leads to a different set of predictions. A comparison of the final results in terms of specificity and sensitivity was done to make the final choice.

##### mRNA module

This module undertakes two jobs, the first one is to map the final candidate set into the gene sequences, and the second is to characterize the environment of the candidate binding sites. After the generation of the *Candidate virtual binding sites *by the GA, a sequencing algorithm is applied with the purpose of mapping the virtual binding sites on the target genes. Here the objective is to check if there are real binding sites on the target genes that correspond, within a certain approximation, to the *candidate virtual binding sites *generated by GA. The approximation is needed because virtual sites are generated in a completely independent way from the real targets, for this reason a perfect match would be too selective. On the other side, the approximation does not apply inside the seed regions where almost a perfect match between mRNA sites and *virtual binding sites *is requested, to reinforce the seed complementarity. From a computational viewpoint, the algorithm computes a weighted hamming distance between each virtual sequence and each possible sequence in the UTR. The target sequences with a *weighted hamming distance *lower than a threshold are called *Candidate Target Binding Sites*. The threshold is set to the hamming distance obtained between any sequence and a site with the seed complementary sequence and at least other 4 matches that is equal to 10 (for a mature miRNA sequence long 22 nucleotides), the sequences with a lower hamming distance are taken into consideration for the next phase. The weighting is performed in order to preserve the similarity in the seed region [5]. To achieve this objective, being *b*_*i *_and *m*_*i *_each base of the virtual binding sites and matching sites (in the gene UTRs) respectively, *N *the length of the binding site and S the seed region (from N-1 to N-8). The hamming distance weighted is computed by the following equation:

(1) $$HD=\overset{N-1}{\underset{i=0,i\in S^{C}}{\sum }}d_{1}\left(b_{i},m_{i}\right)+\underset{i\in S}{\sum }d_{w}\left(b_{i},m_{i}\right)$$

(2) $$d_{1}\left(b_{i},m_{i}\right)=\left\{\begin{array}{cc} 1 & b_{i}\neq m_{i}\\ 0 & b_{i}=m_{i} \end{array}\right)$$

(3) $$d_{w}\left(b_{i},m_{i}\right)=\left\{\begin{array}{cc} 1 & b_{i}\neq m_{i}\\ -1 & b_{i}=m_{i} \end{array}\right)$$

Where the usual hamming distance *d*_1 _is obtained for the region that would bind to the out-seed part of the miRNA. Instead for the miRNA seed binding region a weighted distance *d*_*w *_is obtained to enforce the correspondence in the seed binding region, in this way sequences that are nearly complementary to the seed are privileged.

(4) $$S=\left\{\begin{array}{c} N-1,N-2,N-3,N-4,N-5,\\ N-6,N-7,N-8, \end{array}\right)$$

(5) $$S=\left\{N-1\ldots N-8\right)$$

#### Machine learning Part (Specificity module)

**Figure 4:**
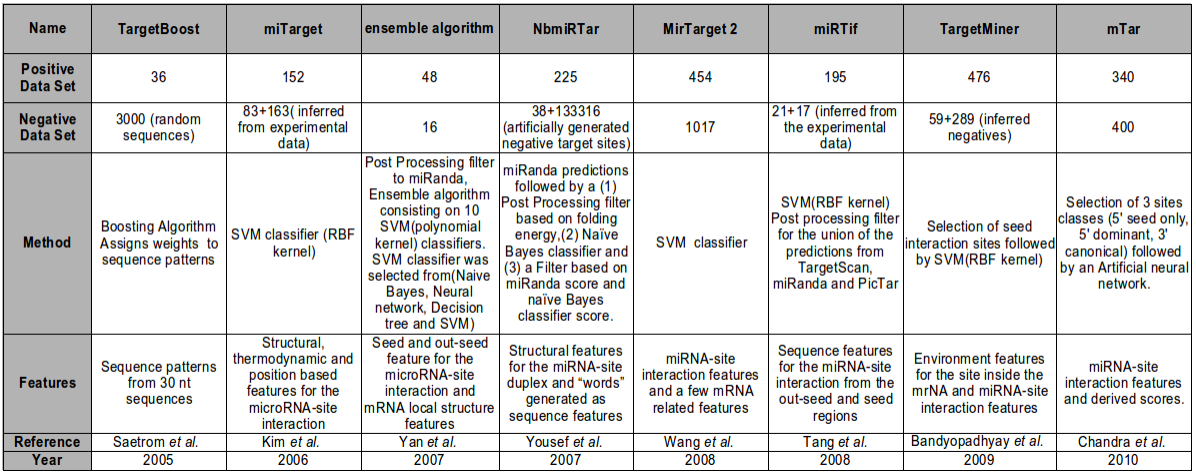
**Table with details regarding miRNA target prediction machine learning tools**.

The *Candidate target binding sites *obtained by the Ab-Initio part are characterized with features related to the site region and the neighborhood of the site (See later in Section Features for a detailed description of these features). The characteristics extracted for the *Candidate target binding sites *are further processed by a machine learning technique to classify the results. In order to choose the appropriate technique to build a classifier we evaluated the techniques employed in previous methods, shown in Figure 4, and the experimental data characteristics. We found that the Support Vector Machine (SVM) was a convenient choice considering its remarkable performance in distinguishing nearest data-points from different classes. The SVM is a computational efficient way for learning in presence of a high dimensional feature space. We characterized the SVM for different kernels (linear, polynomial second order degree, radial basis function) and we performed an optimization of miREE parameters through a 20 cross-validation on Training Set (thus not further considered for the miREE performance evaluation). Details on that are provided in the Results section - miREE parameters characterization. The SVM implementation is based on the *libsvm *package [24].

#### Features

As it was above mentioned the output of the Ab-Initio part are the *Candidate target binding sites*, each one of them was characterized according to features that will be described thereafter. The features extracted from all the *Candidate target binding sites *are given in input to the SVM and consequently they are classified. Each one of the features was selected based on the literature evidence. In general, there are two groups of features used in the machine learning part. One concerns the miRNA-MRE duplex and focuses on the interaction characteristics. The other group, which is related to the environment of the site, takes into account the accessibility to the site itself. In what follows we list all the features used and we number them for reference.

In the first group we selected: (1) Bases unpaired in the seed region; (2) Bases unpaired in the miRNA 13-16 nt [25]; (3) Bases unpaired in the out-seed region; (4) Duplex minimum free energy; (5) seed region contribution to the hybridization energy; (6) out-seed region contribution to the energy. The interaction was divided in the seed and out-seed sections because the seed region is a common factor in a considerable amount of MREs [5]. The base pairing and hybridization energy were considered key factors for the structure stability.

In the second group we characterized the site surroundings by looking at regions of constant length around the site. The features we considered were: (7) AU content in the upstream and downstream neighborhood of the site divided by the mRNA AU content; (8) AU Rich Elements in the entire mRNA (AUUUA pentamer); (9) G motif downstream the site; (10) C motif downstream the site; (11) Free energy for the neighborhood of the site; (12) Free energy in the upstream neighborhood of the site; (13) Free energy in the downstream neighborhood of the site; (14) ΔΔG, difference between the duplex free energy and the free energy of the neighborhood of the site; (15) Position of the site divided by the UTR length; (16) Position in the UTR site (17)UTR length. Additionally the base content in the mRNA sequence normalized (18-21) and the base content in the site (22-25) have also been considered.

The features (7)(8) were selected to characterize the AU content and the presence of pentamers which seem to have a considerable effect in the vicinity of the site as promoter of the miRNA access [26,27]. The attributes (11)(12)(13)(14) were considered to characterize the energetic cost of unpairing the target and make it accessible for the miRNA [28,29]. We extracted (9)(10) additional characteristics from a recent study [30] where a GC-rich RNA motif downstream of experimentally supported miRNA target sites was reported. Finally, according to [2], we considered the relative position of the site as a feature because positions near the start or stop codon are considered more accessible. In the feature extraction procedure the free energy values were obtained using the Vienna RNA package [31].

##### Features ranking

To better understand the impact of the selected features on the accuracy results obtained, a ranking of the features has been done in terms of their individual prediction skills. The results, reported in Table 2, allow to distinguish the most discriminative and informative features. Two metrics were used, namely the *F*_*score *_and a ranking based on the correlation. The first metric represents an index of the capability of each feature to distinguish between the classes used by the ML and it is obtained as: *F*_*score *_= |*μ*_*Positives *_- *μ*_*Negatives *_|/| *σ*_*Positives *_- *σ*_*Negatives *_|, Where *μ *and *σ *are the mean and the standard deviation respectively. Even if the F-score is effective to highlight class separability for a certain feature, it does not take into consideration the mutual information between features [32]. For this reason correlation ranking was also used. This was obtained from the features correlation matrix. In the correlation matrix a generic element *ρ*_*ij *_in the *i-th *row and *j-th *column represents the correlation between the *i-th *feature and *j-th *feature. As such, the *i-th *column express the correlation between the *i-th *feature and the others. Since the objective is to perform a ranking of the features based on the mutual information they carry on, a ranking of the correlation for each column was done (Equation 6). Consequently, the element *rank*_*j *_(*ρ*_*ij*_) represents now the relative position of feature *i *with respect to the correlation with the feature *j*. That is, a value of 1 of element *rank*_*j *_(*ρ*_*ij*_) means that the feature *i *is the one with lowest correlation with feature *j*. Higher values mean higher correlation.

**Table 2:** Features ranking using the cumulative ranking from the F-score and the correlation ranking

Rank	Feature	F-score	Correlation rank
1	normalized mRNA A content	0.3122	1
2	Δ G 3'	0.2727	2
3	ΔΔ G	0.2697	3
4	normalized site A content	0.2733	7
5	normalized site G content	0.2720	6
6	Position in the UTR site	0.3318	15
7	*BU*_*seed*_	0.5770	19
7	normalized AU content	0.3460	17
7	Δ Gopen	0.1987	5
10	normalized mRNA C content	0.1038	3
11	normalized mRNA U content	0.2272	11
11	*Energy*_*duplex*_	0.2175	10
13	UTR length	0.2306	13
13	*Energy*_*seed*_	0.5712	22
15	normalized site U content	0.2070	12
16	Δ G 5'	0.1344	9
16	normalized mRNA G content	0.1036	7
18	AUUUA pentamer presence	0.1345	13
18	Relative position	0.3112	24
20	normalized site C content	0.1564	16
21	*BU*_*miRNA*13-16*nt*_	0.0950	18
22	*BU*_*out-seed*_	0.0670	20
23	G motif downstream the site	0.0599	21
24	*Energy*_*out-seed*_	0.0202	23
25	C motif downstream the site	0.0004	25

(6) $$\begin{array}{c} \left[\begin{array}{cc} \rho_{11} & \rho_{1j}\\ \rho_{i1} & \rho_{ij} \end{array}\right]\Rightarrow \;\;\;\;\;\left[\begin{array}{cc} rank_{1}\left(\rho_{11}\right) & rank_{j}\left(\rho_{1j}\right)\\ rank_{1}\left(\rho_{i1}\right) & rank_{j}\left(\rho i_{j}\right) \end{array}\right]\\ \;\;\;\;\;\;\;\;⇓ \end{array}$$

(7) $$rank\left(\left[\begin{array}{c} \sum_{n}rank_{n}\left(\rho_{1n}\right)\\ \sum_{n}rank_{n}\left(\rho_{in}\right) \end{array}\right]\right)$$

In this transformed matrix, all the rows are summed up to get, for each feature *i*, a global *correlation ranking *with all the other features (Equation 7). This ranking provides a measure of the mutual information carried on by a given feature. To combine both metrics with the purpose of building a final cumulative ranking in terms of the relevance of each feature, first we made a ranking of the features in terms of their F-score, obtaining an F-score ranking. In this case a high rank means a high discriminative capability relatively to other features. Then the two rankings, namely F-score ranking and correlation ranking were added into a final ranking. Using this ranking system, a feature characterized by a high F-score rank (i.e. high discriminative capability) and high correlation rank (i.e. low degree of mutual information) will be in the top positions.

The results are shown in Table 2, where it can be seen that the seed region presents a high discriminative capability having the highest F-scores (*BU*_*seed*_, *Energy*_*seed*_). In addition, features related to the vicinity of the sites such as the AU content, the site position in the mRNA sequence, the cost of unpairing the downstream region (Δ G 3'), ΔΔ G and the A,C,U normalized mRNA content appear in the top positions of the ranking. The presence of 7 mRNA features in the top 10 features highlights the importance of target accessibility information for miRNA target prediction.

## Results

In this section we first describe the methodology adopted for miREE performance evaluation, then we discuss the characterization of miREE parameters. Results about miREE performance evaluation, obtained on the first dataset, are detailed. We also discuss the results of comparative analysis with state-of-art tools, highlighting the contributions of both Ab-Initio and ML modules. Finally, we provide additional evaluations on the remaining two datasets.

### Validation Methodology

In this section we describe the methodology we adopted to evaluate the performance of the proposed method. A complete evaluation methodology must take into account the two steps composing the method. In particular, the performance figures related to the single steps and the whole method must be properly combined.

First, a set of predictions was derived employing the Ab-Initio part. This set is composed of the candidate target binding sites that are predictions for the considered miRNAs on the respective mRNAs (3 UTRs). Consequently the *Experimental Validation Subset *sites were searched into the set of predictions and the records found were selected as input for the second part of the algorithm. These sites represent the predictions of the Ab-Initio part with an experimental basis (Negatives and Positives) that let us evaluate the performance.

Now, there are two sources of possible inaccuracy in the overall prediction performance. The first source is the output of the Ab-Initio step that produces a set of predicted sites (in our case these are called *Candidate target binding sites*), while the remaining sites that are not predicted as target could be either true negatives or false negatives when compared with the experimental data (i.e. the *Experimental Validation Subset*). We refer to these as *TN*_*ab *_and *FN*_*ab *_respectively. Now, only the predictions are further selected by the second part of the method to decide if they are final targets or not, while the non-predictions are not considered in the second step. However, for a thorough validation of the method as a whole, they must be considered in the accuracy computation. Indeed, if the number of *FN*_*ab *_is large, a number of real targets are lost before reaching the second part. Note that this applies not only to our approach, but to hybrid methods in general.

**Figure 5:**
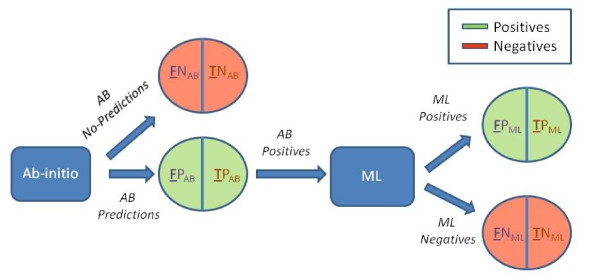
**Block diagram of the validation flow**. Block diagram of the flow followed in order to validate miREE performance, in particular for the first dataset.

The second source of inaccuracy is the Machine Learning (ML) part. Here, positive predictions from Ab-Initio part may be confirmed (positives) or not (negatives). As a result, when compared to the *Experimental Validation Subset*, we end up with true and false positives (*TP*_*ML*_, *FP*_*ML*_) and with true and false negatives (*TN*_*ML*_, *FN*_*ML*_) for the ML part. Figure 5 sketches the validation flow. There, the computational blocks of the method (Ab-Initio and ML) are rectangular boxes while the input and output datasets involved are in labeled circles. Note that only Ab-Initio predictions (AB positives in Figure 5) are passed to the ML part. These predictions may contain true and false positives (*TP*_*ab *_and *FP*_*ab*_), that can become positives or negatives for the ML part (ML positives and ML negatives in Figure 5).

From the previous considerations, the Accuracy (*Q*_*ab*__+__*ML*_), Sensitivity (*SE*_*ab*__+__*ML*_) and Specificity (*SP*_*ab*__+__*ML*_) for the method have been defined as follows:

(8) $$Q_{ab+ML}=\frac{TP_{ML}+TN_{ML+ab}}{TP_{ML}+FP_{ML}+TN_{ML+ab}+FN_{ML+ab}}$$

(9) $$SP_{ab+ML}=\frac{TN_{ML+ab}}{FP_{ML}+TN_{ML+ab}}$$

(10) $$SE_{ab+ML}=\frac{TP_{ML}}{TP_{ML}+FN_{ML+ab}}$$

(11) $$TN_{ML+ab}=TN_{ML}+TN_{ab}$$

(12) $$FN_{ML+ab}=FN_{ML}+FN_{ab}$$

Where TP is the True Positives number, FP is the False Positives number, TN is the True Negatives number, FN is the False Negatives number. The subscript *ab *relates to the Ab-Initio part, while subscript *ML *refers to the Machine Learning (ML) part (Section Method).

In this paper, several tests are presented in order to evaluate miREE performance and miREE comparison with other miRNA targets detection methods, both machine learning and Ab-Initio approaches.

Concerning the datasets employed for evaluation, we used the three datasets described in Section Methods - Data set. Regarding the metric used for evaluation, we measured sensitivity and specificity in all the tests. Precision was also measured where needed for the sake of comparison with public performance data provided by some of the methods; this will be seeing in the following sections. Precision is intended as the ratio of correct positive predictions to all predictions.

The overall method evaluation has been done using the first dataset. Here we adopted a 20 cross validation to get a reliable statistical performance measure. On the other side, for the sake of comparison with other methods, fixed training and test set were used. This was done to make possible the comparison with those methods that do not allow to perform training. To this purpose, for the comparative analysis, the first dataset was split in two parts, 70% for the *Experimental Training Subset *and 30% for the *Experimental Validation Subset*. It is worth noting that there was no overlap between the two subsets neither in terms of miRNA - mRNA interaction sites or miRNAs (that is, the two subsets do not have any record or miRNA in common). The optimization of miREE parameters was performed only on the *Experimental Training Subset *in order to decouple performance evaluation from parameters optimization.

### miREE parameters characterization

To characterize miREE parameters it has been performed a 20 cross validation on the first dataset. In particular, the cross validation was done on the *Experimental Training Subset*. We used this evaluation to determine the optimal SVM parameters (i.e. kernel, cost and gamma) and optimal Ab-Initio module parameters (i.e. fitness function and *α *coefficient in the fitness). We selected the ones providing the best accuracy and the shortest confidence interval with a significance level of 0.05 (*α *= 0.05). In particular, False Negatives *FN*_*ab *_and True Negatives *TN*_*ab *_that characterize the Ab-Initio part have been evaluated to tune its parameters. In order to select the optimal fitness function, optimal *α *coefficient and optimal SVM parameters, the performance of miREE was first evaluated considering (1) data from 7 species and (2) the subset with only the data from the *Homo sapiens*. A detailed description of the optimization procedure is not in the focus of this paper. For this reason, to improve clarity and readability, this description is reported in the Supplementary material (see http://didattica-online.polito.it/eda/miREE/Supplementary_material.pdf). As result of parameters optimization, we obtained the following values: rbf kernel, cost C = 4, *γ *= 2E-1, *fitness*_1_, *α *= 2.

The rbf kernel was then confirmed as a reasonable choice. This is not surprising and justifies its widespread use in state-of-the-art machine learning tools for miRNA target prediction, as shown in Figure 4. The results also pointed out that *fitness*_1 _was the best choice. With *fitness*_2 _we obtained a small decrease in terms of accuracy. It can be attributed to the usage of *Energy*_*seed *_and *Energy*_*out*-*seed *_compared to the *Energy*_*Duplex *_used in *fitness*_1_. We conclude that the energy for the miRNA-MRE duplex seems to be more critical than the individual contributes from the seed and out-seed regions. By comparing with *fitness*_0 _that reached the worst performance, we note that the results point out the relevance of the additional features present in *fitness*_1 _and *fitness*_2_, such as the number of unpaired bases.

### miREE overall performance evaluation

In order to evaluate miREE performance on datasets from different species an extensive validation was done using the *fitness*_1_, the rbf kernel and the other miREE parameters obtained through the optimization just described. As stated early in this Section, in order to show a measure of significance of miREE performance, it has been performed a 10 run, 20 cross validation on the whole first dataset. As a result, we obtained an accuracy of 87.14% +/- 3.46%, with a significance level of 0.05 for data from All-the-species, and an accuracy of 94.77% +/- 2.40% with a significance level of 0.05 for Human data. The results show high values of accuracy with very low fluctuations while changing the training and test sets.

### Comparison with miRNA-target prediction tools - machine learning methods

In order to compare the performance of the method with the state-of-the art tools, first we considered machine learning approaches reported in Figure 4. This comparison was performed on the first dataset (extracted from public databases as described in section Materials and Method - Data set). Note that in this context, adopting a cross validation strategy as done to carry out the overall performance evaluation is not feasible, since other methods do not allow to be trained on external datasets. As such, to compare performance results against a common benchmark, we adopted the *Experimental Validation Subset*. The comparison is still fair, since this validation set and the *Experimental Training Subset *used for training miREE do not have any miRNA:mRNA record or miRNA in common.

In order to make the comparison as complete as possible, we first detailed miREE performance characterization evaluating the contribution of Machine learning part and the contribution of Ab-Initio module. A preliminary study and comparison between the miREE Ab-Initio approach and other Ab-Initio methods (e.g. miRanda, PicTar and TargetScan) is reported in [33]. Nevertheless other tests are reported later in this section to show the effect of the new fitness function and the impact of Ab-Initio part on the overall performance.

Then, we proceeded with the comparison with state-of-the art machine learning approaches. Some of them provided public results related to machine learning method without considering the cost of miRNA:mRNA site detection procedure.

#### miREE detailed performance characterization

We compared the performance obtained by the Machine learning part alone (i.e. the SVM) with the miREE overall performance. Moreover, in order to evaluate the impact of the Ab-Initio part predictions on the Machine learning part and on the miREE overall performance predictions plus the Machine learning part (i.e. the whole miREE method) on the first dataset. In particular, we made the comparison on data from all the 6 species contained in the *first dataset *(namely, *All-the-species*), and data from the *Homo sapiens*, contained in the *first dataset *too. The human species was selected because it has the highest cardinality in the *first dataset *with respect to the other species.

The results obtained for the data from *All-the-species *and *Homo sapiens *are reported in Table 3. We evaluated the differences in the performance obtained using our Machine learning module to classify:

**Table 3:** Accuracy (%) of SVM and miREE obtained on the Experimental Validation Subset

Data from	(1) SVM Experimental	(2) SVM Prediction	(3) miREE Overall Accuracy
	Validation set	Validation set	
All-the-species	83.66	87.76	86
Human	97.09	95.83	93.13

1. the experimental data where precise sites were already provided

2. the output of the Ab-Initio part (without considering in the Machine learning module performance computation the performance of the GA+ virtual sites mapping part)

3. the output of the Ab-Initio part taking into account in the Machine learning module performance computation the performance of the GA+ virtual sites mapping part. We called the performance calculated in this way the miREE's overall performance (see Equation 8).

The first column of Table 3 represents the accuracy obtained on experimental sites classification (see bullet 1 above). Note that this is an ideal case because a prior selection of the sites to give as input to the Machine learning module is necessary. The second column represents the case where the SVM classifies the output of the Ab-Initio part without taking into account its performance (see bullet 2 above). The performance of the Ab-Initio part, that is not here evaluated, concerns the predictions that were not made by the Ab-Initio part (i.e. False Negatives and True Negatives). The third column represents the overall accuracy where the contribution of the Ab-Initio part is taken into account (see bullet 3 above). It is important to note that in most of the state-of-the-art hybrid systems only the accuracy related to the machine learning technique is reported (i.e. see (column 1) in our case). However, also the accuracy of the Ab-Initio part must be considered to provide a global accuracy evaluation of the method in its typical use. For the sake of clarity, we report both results, nonetheless the overall accuracy is considered in this paper as the key factor that characterizes miREE performance.

Looking at the differences between the first and the second column of Table 3, we can see that the performance of the SVM slightly decreases, as expected (except for all-the-species records that we will discuss later). However, the gap between the two achieved accuracies is quite small meaning that the Ab-Initio part is a robust step for the selection of the candidate sites to give as input to the Machine learning part. Another reason for this small gap is that the SVM is able to filter out the sites erroneously identified as positives by the Ab-Initio part. Also the gap between the accuracies in column 2 and 3 is very small meaning that the Ab-Initio part is definitely able to correctly classify the negative records. Concerning the difference between column 1 and 2 for all-the-species records, a higher accuracy is obtained using miREE because the Ab-Initio part correctly identified a large number of true negatives (larger than the false ones) filtering out some bad sites before giving them to the SVM. In fact the gap between column 2 and 3 is in this case particularly small. It is actually smaller than the one concerning human records. On the other side, in this last case, the Ab-Initio part is less capable to correctly distinguishing between true and false negatives and in fact the performance decreases from column 1 to column 3, and the gap between column 2 and 3, although small, reveals a major contribution in the overall performance of Ab-Initio part errors.

In order to evaluate the effectiveness of the miREE negative expansion method, we generated negatives to be used as training using alternative strategies. To do so, we implemented these methods to devise their expanded negative records. The approaches we used for comparison are miRTif [11] and miTarget [12] negative expansion strategies. We did not consider NBmiRTar [13] and TargetMiner [14] for the comparison because in both cases the negative records they generate have not direct relationship with structural properties.

Once the expanded negatives have been obtained, we tested the performance of three different versions of miREE, each one using the same positive set (experimental) and a different negative set for training the ML (machine learning) part. Note that for test, we used only experimental positives and negatives without expansion. The results of this comparison are shown in Table 4 for data from *All-the-Species *and from the *Human*. Results demonstrate that the strategy used by miREE to expand the negatives outperforms state-of-the-art strategies.

**Table 4:** Overall accuracy obtained using different expanded negatives constructed for the All-the-species records and the Human records in the first dataset

Expandend Negatives	Overall accuracy (%) miREE *All-the-species*	Overall accuracy (%) miREE *Human*
miREE's expanded negatives	86	92
miRTif's expanded negatives	72	85
miTarget's expanded negatives	83	88

Moreover, in order to make an evaluation of the impact of the Ab-Initio part and in order to demonstrate the advantages of having an integrated tool, we compared the performance of miREE with the one obtained using a different algorithm in pipeline with our SVM. This alternative approach might still enable the identification of candidate target sites to input to the SVM. miRanda [6], as widely known and used tool, was selected for this test.

**Figure 6:**
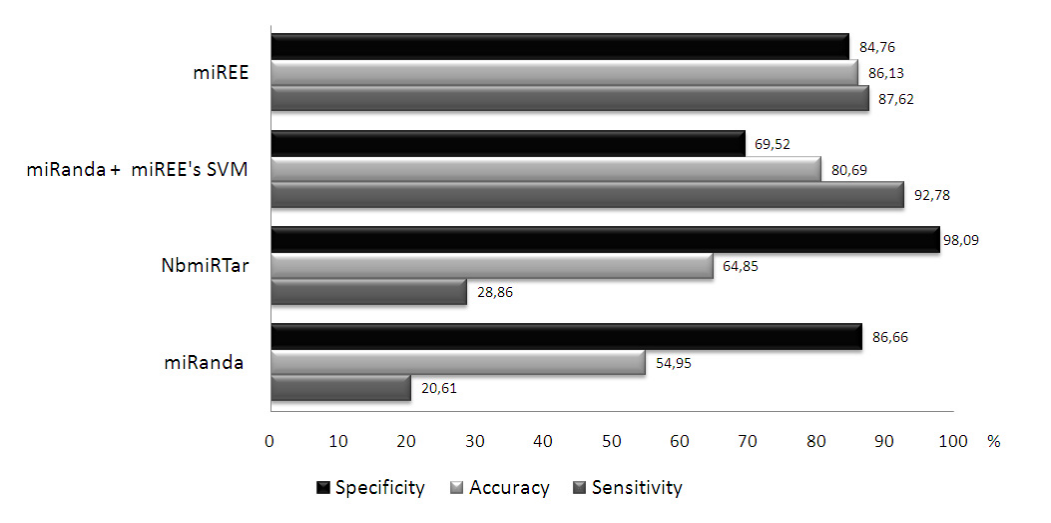
**NbmiRTar, miRanda (default settings), miRanda (no filter)+ SVM and miREE performance (%) on the predictions over the All-the-species records in the first dataset**.

The obtained results are reported in Figure 6. The performance obtained with miRanda + SVM compared to miREE shows that the coupling of the Ab-Initio part with the SVM really improves the results. This because miREE overall performance is characterized by a very good trade-off between sensitivity and specificity, moreover it is remarkable compared to miRanda + SVM performance. Note that in this case we used miRanda without any type of restrictions and for this reason the sensitivity is high. However, default settings of miRanda imply several restrictions that affect its sensitivity. To highlight this, in the same figure the performance of miRanda with the default restrictions is also presented

We also took into consideration the results obtained with NBmiRTar [13], in such a way, to compare miREE with a hybrid but not integrated tool that uses miRanda to identify the possible candidate binding sites. All the results obtained on the *Experimental Validation Subset *are reported in Figure 6.

It is worth to briefly describe NBmiRTar. It uses a Naïve Bayes approach to classify the output of miRanda, first it uses miRanda with a less restrictive filter in terms of energy, then it uses two additional filters. The first one is based on the score obtained using miRanda (dependent on the base paring), and a second filter based on the Naïve Bayes Score. As it can be seen in Figure 6, NBmiRTar has a better performance compared to miRanda (with default settings), indeed its sensitivity is higher and at the same time its specificity and accuracy are higher. Nonetheless, both NBmiRTar and miRanda are characterized by poor balancing between sensitivity and specificity. In addition, the results obtained with miRanda + miREE's SVM compared to NBmiRTar show that the filters used inside NBmiRTar improve the specificity, but the sensitivity significantly degrades. Finally, comparing miRanda + miREE's SVM and miREE, the relevant role played by miREE's Ab-Initio part becomes evident. Its integration with SVM provides a better trade-off between sensitivity and specificity. When miREE's SVM is used after miRanda predictions, the specificity degrades because the SVM is unable to correct some of the false positives predicted by miRanda. On the other side, considering the expansion of the negatives performed through the genetic Ab-Initio part, the SVM is able to recognize some of the wrong predictions thus improving the overall specificity of the integrated method, without impacting sensitivity, which remains high.

#### Comparison with previous Machine learning approaches

For the comparison with the machine learning tools we first provide a summary of the main characteristics of the tools in the Figure 4. In particular for each machine learning approach employed, the negative and positive records are highlighted. Concerning the comparison with the methods reported in Figure 4, we have to provide some preliminary consideration. In order to make a fair comparison, we could not refer to their published predictions or measures of performance (i.e. AUC, Accuracy, Sensitivity, and Specificity) because these data are obtained on different datasets.

In order to make an objective comparison we thus used the publicly available methods to retrieve the sets of their predictions on the *Experimental Validation Subset*. These methods provided correct predictions, as well as false positives and false negatives. Thus, using their predictions we could directly compare their results against miREE. We were able to carry out this comparison with four tools: miTarget [12], NbmiRTar [13], TargetMiner [14] and mTar [16].

**Figure 7:**
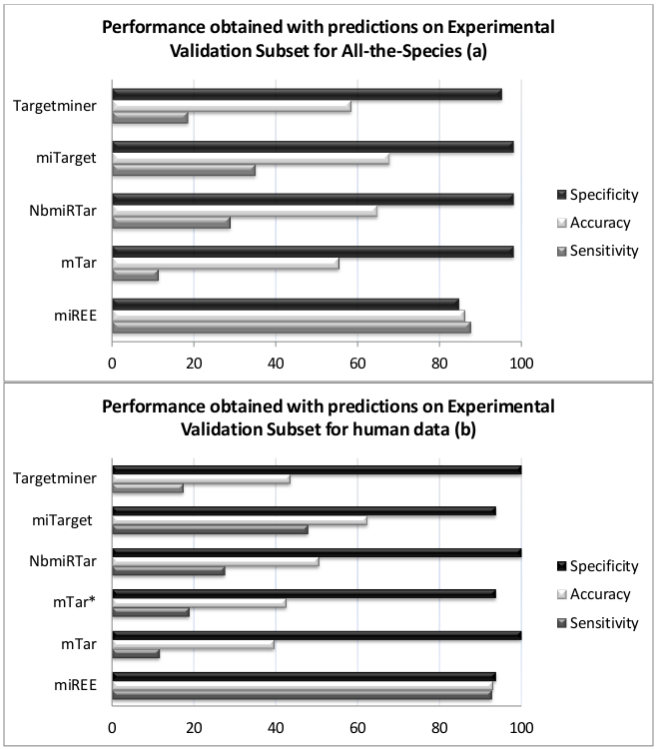
**7(a) and 7(b) miTarget, NbmiRTar, Targetminer, mTar and miREE performance (%) on the predictions over (a) data from All-the-species records in the first dataset (b) data from the Human records in the first dataset**. Figure 7(a) and 7(b) show the performance (%) achieved (in terms of accuracy, sensitivity and specificity) of the proposed method against other methods.

We used the sets of predictions obtained from each one of these 4 tools to validate the performance, having the *Experimental Validation Subset *as evidence. The results obtained on this subset highlighted for *All-the-species *records and for *Human *records are reported in Figures 7(a) and 7(b). Globally, miREE has the highest accuracy equal to 86% considering *All-the-species *(and about 93% on *Human *data), notably higher than the best of the other tools achieved by miTarget equal to 68%. Even though the specificity is slightly better for the other tools, their sensitivity is notably reduced and as a consequence there is disequilibrium in their performance. Instead miREE keeps a notable balance between specificity and sensitivity, as it can be seen. The same considerations can be done for the results obtained on the *Human *data, nonetheless the differences between miREE's sensitivity and specificity balance with respect to the other tools' disequilibrium are enhanced in this case.

In addition, concerning the Machine learning performance, we performed where possible the training of the Machine learning part using the same *Experimental Training Subset*. Unfortunately only mTar offers this possibility. We refer to mTar* as the trained version of mTar as shown in Figure 7(b). The former performed slightly better than the latter, without reaching the accuracy of miREE. It is worth noting that mTar was originally trained on data from the human; in order to evidence the differences dependent on the training dataset, the new training and evaluation was done on human data. It can be said that changes in the training set of mTar did not affect significantly its performance. As a result differences in the accuracy with respect to miREE can be attributed to differences of the methods.

##### Comparison with prediction methods on their datasets

A different methodology has been used to carry on the comparison with Targetboost [10], miRTif [11] and the Ensemble algorithm [15] because it was not possible to obtain the predictions of these tools due to internal restrictions. As we cannot use the first dataset as for the other methods, we are forced to run our tool on their dataset.

TargetBoost is an available machine learning tool where the miRNA data can be given in input, but the mRNA sequences cannot be changed and they are from the *C.elegans*. In order to perform the comparison, we considered all the experimental data with evidence for this species. On the Targetboost experimentally validated data we obtained a sensitivity equal to 87.5%; only the sensitivity was evaluated considering that in Targetboost data only Positives had experimental evidence. Additionally we used TargetBoost and miREE to make predictions for 2 miRNA-mRNA validated interactions obtained from the study [34] but not present in TargetBoost dataset. Targetboost was not able to predict any of the sites, while miREE predicted both.

Regarding the comparison with miRTif and the Ensemble algorithm, it is worth noting that: (1) the tools are not functionally available for the public use at the moment of writing; (2) their datasets were available; (3) their published results were related only to the Machine learning part performance directly on experimental data sites. Thus for the sake of completeness, we performed a comparison between these tools and the miREE machine learning part that was trained with their experimental data, respectively. The obtained results were compared with the publicly available results for both methods. It is worth noting that we performed a 10-fold-cross-validation to obtain the accuracy like miRTif and the Ensemble algorithm did. To achieve this goal, the features described in Section Features were extracted from the miRTif and Ensemble training datasets respectively and were used separately to train the SVM with the radial basis function. The results obtained using a 10-fold-cross-validation accuracy can be seen in Table 5.

**Table 5:** Accuracy (%) 10-fold-cross-validation obtained with training miREE's SVM using miRTif and Ensemble training set

Data set	miRTif	Ensemble Algorithm	miREE's SVM
miRTif training set	81.97	-	89.56
Ensemble training set	-	82.95	83.92

The improvement obtained with the machine learning part of miREE is attributed to the quality of the features extracted by the methods under comparison. Both miRTif and the Ensemble algorithm are presented as post-processing filters to Ab-Initio algorithms, both of them are capable of extracting features regarding the miRNA-site duplex and external features of the mRNA, but they are unable to extract characteristics of the site vicinity.

### Performance evaluation on additional datasets

In this subsection, to achieve a more complete characterization of miREE performance, we evaluate its accuracy using two additional datasets, namely PAR-CLIP [21] and *indirect validated *dataset [20]. PAR-CLIP has been selected because this data has been obtained using an innovative experimental procedure which is based on direct validation, even if there is not detailed information about miRNA-mRNA target pairs. On the other side, the *indirect validated *dataset contains only indirect validations of miRNA targets. Even if this is not suitable to our approach which is heavily based on structural features of the binding site, we did this evaluation to enable the comparison with various Ab-Initio methods that has been tried on the *indirect validated *dataset in [22].

#### Performance evaluation on indirect validated dataset

Various Ab-Initio methods have been compared in [22] on the *indirect validated *dataset [20], such as miRanda [6], TargetScan [7], Pictar [8], Diana [35], EIMMO [36] and RNA22 [37]. They mainly based the prediction in recognizing seed complementary sequences in the 3'UTRs, and they select among the chosen sequences according to the degree of conservation or/and hybridization energy employing different strategies. A different approach was done in PITA [28] which incorporates accessibility features. A more detailed description of the Ab-Initio methods can be found in [3,22].

A comparison with previous Ab-Initio methods for microRNA target prediction was done on data obtained through indirect validation extracted from [20]. This dataset consists of measured changes of protein levels and quantified mRNA levels after the transfection of 5 miRNAs in Hela Cells. In total there were 15806 associations for potential gene:miRNA interactions. In the original paper [20] proteins with an associated *log*_2_(*fc*) < -0.1 were considered as significantly affected. In a posterior review of microRNA target prediction algorithms a stricter definition was taken into consideration: Proteins with a *log*_2_(*fc*) < -0.2 were considered significantly affected by miRNAs that is transcriptional-targets. For the sake of clarity we present miREEs performance results considering both thresholds *log*_2_(*fc*) < -0.1 and *log*_2_(*fc*) < -0.2. We compared miREE against the most common Ab-Initio methods, however the performance of the other methods was obtained from [22] where the threshold *log*_2_(*fc*) < -0.2 was taken into consideration.

It is worth mentioning that giving the *indirect validated *dataset the specificity cannot be obtained because there is no experimental evidence that a protein with *log*_2_(*fc*) higher than the imposed threshold will not be transcriptional target. Thus the dataset does not give support for negative data. Instead we extracted the *sensitivity *and the *precision *as in equations (13) and (14) respectively.

(13) $$Sensitivity=\frac{Number\;of\;genes\;predicted\;from\;SS}{Number\;of\;Genes\;in\;SS}$$

(14) $$Precision=\frac{Number\;of\;genes\;predicted\;from\;SS}{Total\;number\;of\;predictions}$$

Where the *selected set SS *corresponds to the genes with associated protein expression *log*_2_(*fc*) smaller than the imposed threshold (-0.1 or -0.2). It is worth noting that the positive records obtained with indirect experimental techniques include both direct and indirect targets. The latter are targets that are not directly affected by the miRNA but rather they are affected by another target of the miRNA impacting their expression.

**Figure 8:**
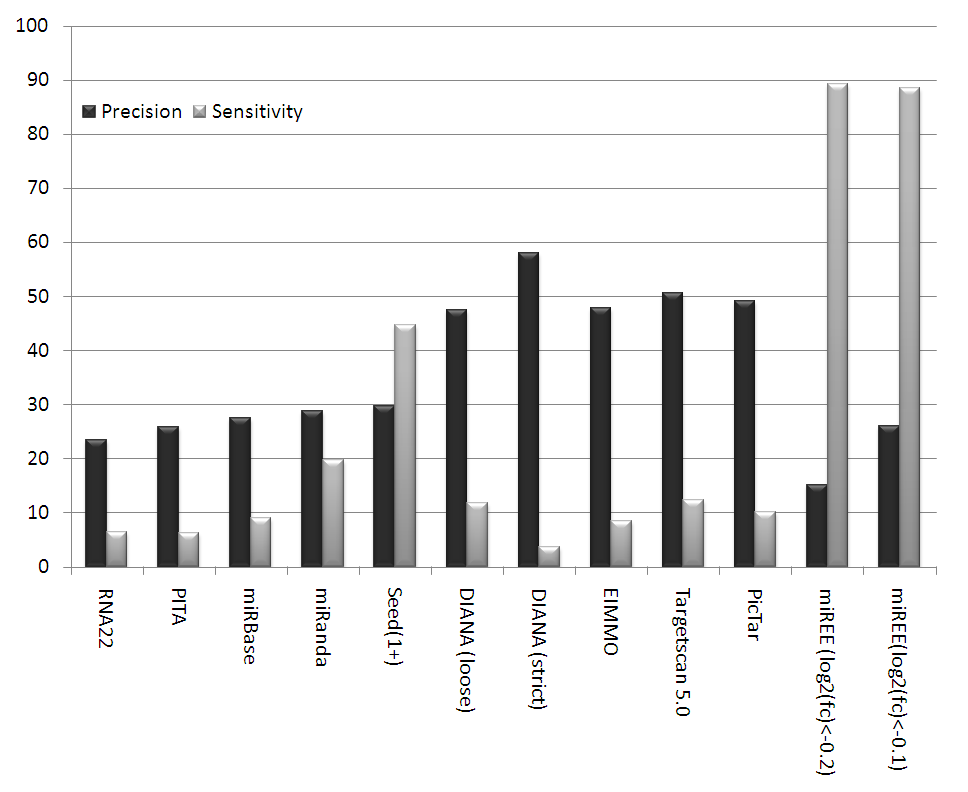
**Comparison of miREE sensitivity and precision (%) against nine Ab-Initio methods on the *indirect validated dataset provided by ***[20]. Figure 8 shows the performance achieved (in terms of sensitivity and precision (%)) of the proposed method against nine Ab-Initio methods on the *indirect validated *dataset.

In Figure 8 it is shown the performance of the Ab-Initio and miREE methods at their working points, which are with their default settings. As it can be seen in Figure 8, miREE is largely the most sensitive method, however its associated precision is one of the lowest. Nonetheless miREE's precision is comparable to tools such as miRanda [6], PITA [28] and RNA22 [37] and its associated sensitivity is more than 4 times the sensitivity obtained with these tools. The more precise tools DIANA, EIMMO, TargetScan and Pictar have a precision near to 50% but the levels of sensitivity do not pass the 11%. The poor sensitivity of tools like Pictar, TargetScan and DIANA(strict) that intend to capture targets that are significantly conserved could be attributed to the fact that the dataset is composed of both conserved targets and also non-significantly conserved targets. This low sensitivity makes visible their strong restrictions regarding conservation, and the fact that candidate targets from an indirect dataset can be not necessarily strongly conserved.

It should be noted that it is not well established where the threshold should be set to differentiate a target from a non-regulated gene. In fact the number of False Positive and True positives depends on the threshold values *log*_2_(*fc*) < -0.2 or *log*_2_(*fc*) < -0.1. As a consequence, miREE precision varies depending on the threshold however its sensitivity remains almost constant.

However, since most of the tools in Figure 8 operate at very low sensitivities, the comparison with miREE is not fair.

**Figure 9:**
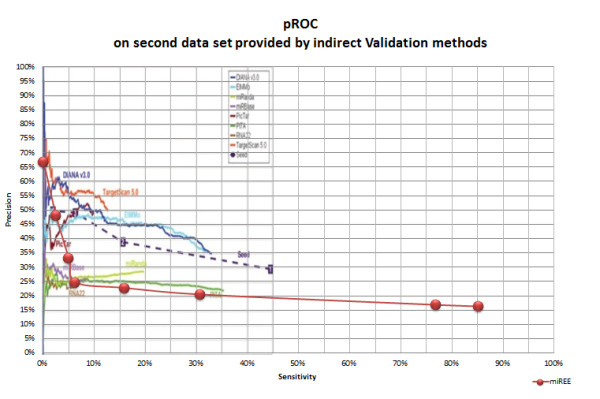
**pROC obtained for the Ab-Initio approaches in the indirect validated dataset. The original figure taken from **[22]**has been modified to show miREE points of precision vs sensitivity**.

**Figure 10:**
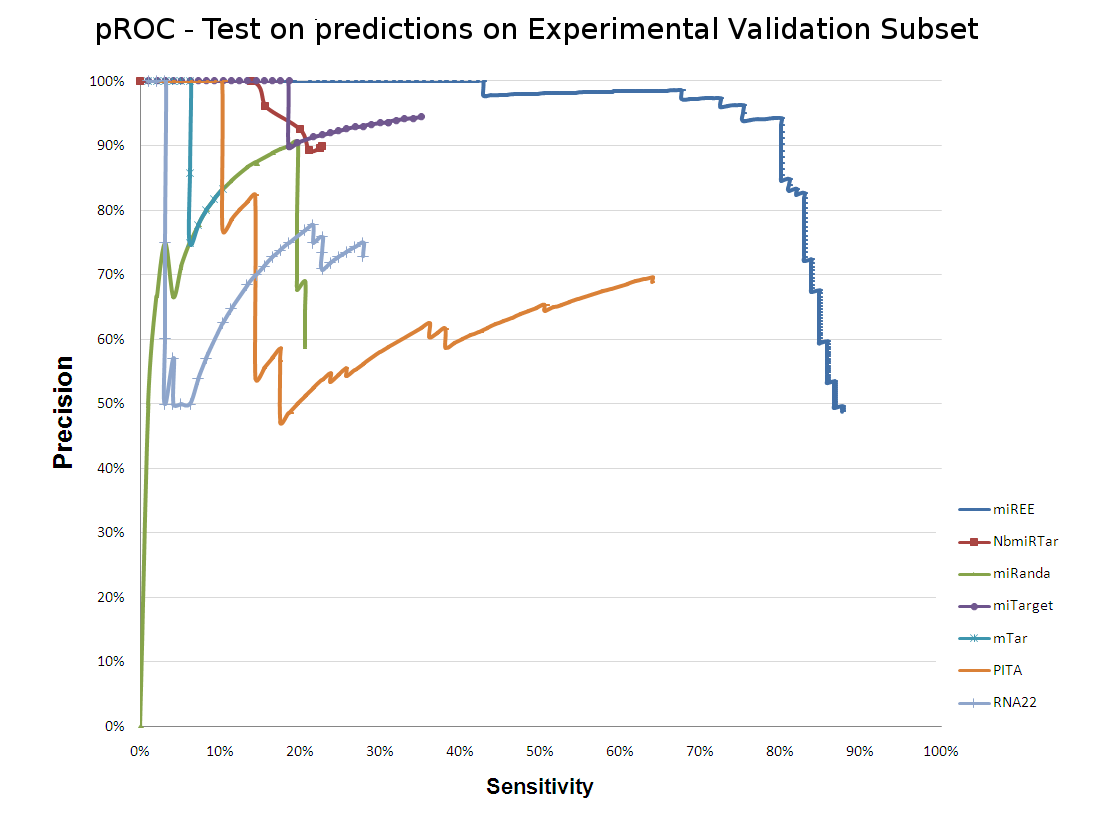
**pROC obtained for various Machine learning and Ab-Initio approaches in the Experimental Validation Subset**.

In Figures 9 and 10 we thus show the pROCs where various machine learning and Ab-Initio methods are compared using the indirect validated dataset and the direct *Experimental Validation Subset *(first dataset), respectively. For the indirect dataset the pROC consists of different points of operation of miREE overlapped with the pROC presented in the review of microRNA target prediction approaches done in [22]. While, for the *Experimental Validation Subset*, we present the pROC obtained by using the machine learning approaches and the three available Ab-Initio approaches. As it can be seen in the second pROC, miREE is considerably precise in the detection of direct targets.

Before discussing Figure 9 focusing on low sensitivity regions, we first recall the results in Figure 8, where it is evident that 6 out of 10 algorithms are characterized by operating points with a sensitivity lower than 10%. In particular, RNA22, PITA and DIANA (strict) work at operating points characterized by sensitivity around 5% - 7%. By comparing miREE performance with other methods at comparable operating points, the former shows better performance (see Figure 9). In fact, for a sensitivity of 5% miREE is characterized by a level of precision higher than 33%. At this operating point miREE precision is better than the precision levels obtained by RNA22, PITA, miRanda and miRBase. This is also true in their whole sensitivity range. Note that, in particular, the sensitivity of RNA22 and miRBase does never reach more than about 7% and 9%, respectively, and their corresponding precision values are around 23% and 28%. Instead PITA operates by default with a sensitivity of 7% and a precision of about 27%. Thus, by setting the operating point of miREE according to a sensitivity value comparable to those of most of the other methods, we reach better performance.

Moreover, we want to highlight, still in figure 9, not only the level of precision of miREE but also that the other tools are characterized by a much lower maximum sensitivity values. In fact, tools with highest sensitivity (excluding miREE) reach a maximum level of sensitivity of about 35% (and equivalent precision). Nevertheless miREE is the only one that achieves sensitivity higher than 80%.

The difference in terms of precision between the test on first dataset and the test on high-throughput *indirect validated *data is mainly due to two expected reasons: first of all the biological assumption under these two tests is completely different. In the first case we are testing the ability of the method to detect direct miRNA targets, which are those targets that are characterized by structural affinities with miRNA molecules. We can expect here to see the best performance of miREE because it is designed for detecting structural affinities between single miRNA and mRNAs. While the second test aims at identifying a set of mRNAs functionally regulated by a set of miRNAs. This implies that not only direct miRNA targets but also indirect targets can be found. Indirect target are those ones that are in the regulative pathways but that could not present structural affinity with miRNAs. So it is pretty clear that in this second test we can expect worse performance with respect to the first test.

The second reason why we can expect a difference in the performance between the first and the second test relies in the nature of high-throughput experiment provided by [20] that we use for our second test. We believe in fact that further considerations should be taken when working on data validated with indirect methods. This dataset, as many others in the literature, is obtained measuring gene expression difference before and after miRNA over-expression. Actually, in miRNA over-expression experiments there might be targets that even though are affected by miRNA over-expression, do not show a high degree of down-regulation due to factors like the saturation of the miRNP complex. In fact, miRNA over-expression can potentially saturate RISC complexes and displace other endogenous miRNAs [38] and consequently cause low affinity target sites to appear functionally important [39]. For this reason there can be real targets not showing a high degree of down-regulation. These data are thus considered as negatives for a specific threshold, hence they could be wrongly identified as false positives. This fact impacts directly on the precision. Lower precision means that there are in the prediction many false positives, but in this kind of experiments we are not really sure if false positives are correctly classified as false [38,39]. Thus, the measure of the precision is, in this kind of experiment, not at all robust and thus it is not fair to be used as evaluation criteria.

Besides the criticalities we just mentioned about this kind of *indirect validated *data that make precision evaluation not consistent, the comparison with Ab-initio methods in Figures 8 and 9 is interesting because it reflects the impact on the performance of strict/loose feature filtering. In particular, we refer to filters (namely, restriction) that impose a strict requirement the presence of some features neglecting the possibility of feature compensation (i.e. the evaluation of overall features contribution). Due to this filtering, most of the other algorithms improve their precision, but they loose on sensitivity.

By looking at experimentally validated data and searching for the presence/absence of features, we note that even though there are features frequently present in the identified recognition elements, there is not a single feature that is present in all of them, not even the complete seed pairing. For this reason we do not assume any predefined restriction when applying feature filtering. miREE, instead of using restrictions uses the SVM trained on the experimental training set. In this way it attempts to automatically compute the overall features contribution present in validated samples and allows feature compensation. For example, most of the algorithms that are precise impose the seed pairing restriction. We are aware of the importance of the seed feature; in fact we do consider and reinforce the seed search in our algorithm because it is one of the main features that characterize mREs (microRNA recognition elements). However, we want to highlight the possible issues caused by imposing the presence of a specific feature. In a recent study about HITS-CLIP [40] around 27% of the sites identified for the Argonaute were not identified using a seed based strategy. Ignoring this issue conduces to lose a significant fraction of the targets.

We use the first dataset to provide also measure of precision in order to show that miREE is not affected by precision issues. We performed a test also on CLIP data, latter in this Section, and we show also here a high specificity. In fact, the miREE selected operating point works well in these two reliable datasets, the first dataset and the PAR-CLIP dataset. It allows a remarkable performance on both experimentally validated datasets in terms of both Sensitivity and Specificity.

#### Performance on PAR-CLIP data

To further validate miREE, a performance test was done on the second additional dataset obtained with a recent high-throughput technique CLIP (Crosslinking Immunoprecipitation). For this aim, it was extracted a subset from the data obtained in a recent study [21] using the PAR-CLIP method (Photoactivatable-Ribonucleoside-Enhanced Crosslinking and Immunoprecipitation). The subset is composed of 596 identified binding sites with 6-mer seed matches for 11 miRNAs. Where these 11 miRNAs correspond to the miRNAs that are in the top 25 expressed miRNAs, and that are not part of the training set. In this order of ideas, miREE was used to make predictions for those 11 miRNAs and the results were assessed using the subset obtained with the PARCLIP method. For the 11 miRNAs miREE was able to identify 429 binding sites from the 596 experimentally identified binding sites, which means miREE obtained a sensitivity of about 72%, confirming the capability of the proposed method to distinguish binding sites.

Note that the CLIP repository [21] also provided information about expressed transcripts that were not associated to any experimentally recognized binding site (namely Un-CLIPed transcripts). Sites in these transcripts may represent not-targets for the top expressed miRNAs. However, there is lack of experimental evidence that all of these actually represent real negative targets. In fact, it is possible that sites that do not have any associated CLIP cluster may contain binding sites that are not rich in U content and sites devoid of U content and thus that cannot be captured using 4SU-based crosslinking, the one used for transcript labeling by PAR-CLIP method. These transcripts could contain binding sites that have been mistaken as negatives [21]. We know that these types of sites are a minority. Nonetheless this possibility should not be neglected. For this reason we did not consider them to enrich the set of negatives used in the training set.

However, in order to complete our evaluation of miREE performance we considered also these negative sites to perform a measure of specificity. Thus, we added to our PAR-CLIP dataset 952 sites from the Un-cliped transcripts that do not mediate regulation (thus negatives sites) and we computed the specificity. As a result, we obtained a specificity of about 64%. Since, as above mentioned, we are not sure that all the negative sites are in fact real negatives, we could expect a higher specificity. Nevertheless, even without this correction, this result highlights the performance of miREE in terms of specificity and thus its usability.

## Discussion

For miRNA target prediction, one of the main drawbacks of the machine learning approaches is the lack of information regarding the true negatives. This is a critical factor considering that these approaches are intended to reduce the number of false positives. Indeed, this drawback impacts the capability of distinguishing between a negative and a positive sample.

One of the first approaches to expand the negatives records was proposed in [41] and used in Targetboost. It consisted in the generation of random sequences using the background base frequencies found in the UTRs. The random sequences were labeled as *negative *examples. In a similar way, NBmiRTar suggests to consider as false positives a set of 133316 miRanda predictions for 100 artificial mature miRNA sequences generated randomly. However these proposals have some limitations: (1) they lack experimental support; (2) the random sequences may have very different characteristics from the miRNA recognition elements, as a result they do not represent a good training for distinguishing positives against negatives compared to the real samples; (3) the generated sequences can interact with miRNAs, so that positives and negatives are confused.

Targetminer expanded the set in three stages. In a first stage, they identify over-expressed miRNA-mRNA pairs in specific tissues, using miRNA and target microarray data. In a second stage, pairs identified are filtered by comparing the expression of the selected non-target with expression levels of the other genes in an independent microarray data. In a third stage, the mRNAs that have an acceptable miRNA-(mRNA site) duplex stability or have a conserved site for different species are filtered out. In the third stage this method requires to filter out the sites with characteristics similar to the experimentally supported MREs, thus introducing the risk of lowering the quality of the data. In practice, this technique leads to a separation of the features of the negative samples from the positives. It is worth mentioning that the third stage is necessary to avoid inconsistencies produced by the uncertainty coming from the correlation of two independent microarray experiments and the indirect gene relations.

Two alternative approaches are proposed in [12] and [11]. The first one (adopted in miTarget) defines a set of inferred negatives coming from the observation that after the deletion of the target sites in some target mRNA sequences, no repression was experimentally observed. Authors collected the examples with more than 4-mer matches in their seed. They gathered 163 samples for two targets from the *Caenorhabditis elegans*. In the second approach (adopted in miRTif), binding patterns on known negative interactions are predicted using RNAHybrid [42]. In both cases, the negative sets are experimentally supported and they are composed of sites having characteristics close to miRNA interaction elements (i.e. more than 4-mer in the first case, RNAhybrid predicted interactions in the second case).

For this reason, we improved their strategies employing our Ab-initio part search engine to make predictions on non-regulated genes. Those genes are obtained from experimental data and represent targets that are not translationally repressed or transcriptionally downregulated by a specific miRNA. The predictions performed by Ab-Initio part on these targets were labeled as negatives and used to enrich the negative set. Our procedure for the expansion of negative sites is remarkable for three main reasons. First of all, the negative records used for the SVM training derive from sites extracted from non-regulated genes with experimental support. Second, these sites are designed in order to facilitate an accurate definition of the SVM hyperplanes that divide the classes. In fact, the negative sites obtained as miREE Ab-initio predictions on experimental negative data, have structural characteristics similar to the mREs. It is worth noting the importance of this fact considering the characteristics of a SVM. In particular, to proper train a SVM classifier it is crucial to have a training set composed of samples located in the borders between classes in order to accurately define the hyperplanes that divide the classes. For this reason, a negative set composed of samples with experimental support and structural characteristics similar to mREs is fundamental.

Finally, the improvement with respect to other approaches relies also in the fact that the use, during the SVM training, of the negative sites wrongly identified by the Ab-initio part and given in input to the SVM as non-targets makes the SVM able to identify and filter out some erroneous predictions made by the Ab-initio module.

We believe that this negative expansion technique is a relevant contribution to the good trade-off between specificity and sensitivity metrics evidenced by the results of the comparative analysis as it can be seen in Figure 7 and on the PAR-CLIP dataset. Besides the remarkable absolute accuracy score, this trade-off represents a distinguishing aspect of miREE with respect to other tools, which in general fail to combine the contrasting requirements of reducing the number of false positives and achieving correct predictions of the true positives.

Concerning the comparative analysis shown in Figure 7, a more detailed discussion is required about the choice of the validation set, as this is key for a thorough and fair comparison. For all the methods a validation set composed of positive samples extracted from public databases has been used. The negative set, on the other side, has been generated using the Ab-Initio prediction method previously described.

However the accuracy gap between miREE and the other methods cannot be attributed to the strategy we used to generate negative data, as they were able to recognize most of them (see a high specificity). Rather they failed on true positive recognition.

Furthermore, miREE presented a remarkable sensitivity in two additional datasets, referred as the *indirect validated *and PAR-CLIP datasets. In order to improve the performance of miREE on indirect validation data, the induced level of expression should be integrated to miRNA target prediction methods as an additional source of information. While, concerning the test on PAR-CLIP data, the obtained sensitivity and specificity confirms the capability of miREE to make an accurate prediction of miRNA targets balancing the sensitivity and the specificity. That allows identifying a great number of real targets without having an explosion of false positives. At the same time, this avoids to lose too much real targets in order to keep down the number of false ones. In the PAR-CLIP data are obtained through an improved method for isolation of segments of RNA bound by RBPs (i.e. RNA-binding proteins) or miRNPs (i.e.microRNA-containing ribonucleoprotein complexes), the PARCLIP (Photoactivatable-Ribonucleoside-Enhanced Crosslinking and Immunoprecipitation). To facilitate crosslinking, 4-thiouridine (4SU) were incorporated into transcripts of cultured cells. That allowed identifying the binding sites of cellular RBPs and miRNPs by scoring for thymidine (T) to cytidine (C) transitions in the sequenced cDNA. Thus, we cannot properly call this kind of experiment *direct validation *because the RBP binding sites were detected instead of the single miRNA:mRNA interaction. However, we considered PAR-CLIP data as a direct validation and the results confirmed the capability of miREE to deal with direct miRNA targets and PAR-CLIP data too because it takes into account also the characteristics of the site surroundings (both downstream and upstream).

Given the complexity of miRNA-mRNA interactions and miRNA downstream effects, we considered relevant to select the miRNA target candidates based not only on a single criteria but rather finding the optimal combination of features that maximizes the resultant accuracy. In fact, the use of the GA enables to obtain virtual sites combining several features and, more important, optimizing the combination of them. Note that the combination of features is included in the fitness function that was optimized based on the accuracy obtained on experimental data. In addition, using the GA allows recovering sites where the feature compensation is present (that is an evaluation of features contribution). It is well known that features compensate between each other [25,5]. For example, the 3' miRNA pairing compensates for an incomplete seed pairing. While a search based on the seed complementarity would not recover the sites with 3' compensative pairing, the GA is able to recover these sites. The use of the GA output set for homologous miRNAs and the study of the differences among the most frequently mapping candidates in different species, could lead to identify variations in the enrichment of structural characteristics of the targets among different species. Moreover, the GA lends itself to be easily extended with new features. We consider that the use of the GA introduces flexibility and the ability to recover a most accurate set of sequences in comparison to a search based on mutations. In addition, the GA enables to obtain a set of negatives suited to train a classifier such as the SVM.

Finally, the characterization of the ML part obtained by evaluating the feature relevance with respect to the final accuracy proves that site accessibility plays a key role in target recognition. The fact that features regarding the out-seed region and the duplex minimum free energy do not have a higher score is related to the technique we adopted for the expansion of the negative records, where sites in non-regulated mRNAs were selected based on a high duplex stability. For this reason, the negative records and positive records have similar properties as far as the miRNA-site stability is concerned. On the other side, the features regarding the site out-seed region play a relevant contribution to the selection process in the context of samples with low duplex stability.

## Conclusions

In this paper we presented a hybrid integrated approach for microRNA target prediction composed of an Ab-Initio and a machine learning (ML) modules. The proposed approach is aimed at addressing two main limitations of state-of-art approaches. First, a relevant issue regarding approaches integrating a ML part is the lack of negative data given the experimental data available, which was addressed through a GA approach for expanding the negative set. Second, the number of false positives is consistently reduced through the integration of the GA approach and the SVM module, without losing the sensitivity that characterizes the Ab-Initio part. miREE is able to obtain a remarkable balance between sensitivity and specificity, compared to the state-of-the-art. It is critical to identify microRNA targets without introducing uncertainty in the reliability of the predictions. For this reason, recognizing the experimental targets publicly available is mandatory. Besides miRNA-MRE duplex stability, the accessibility characteristics provide relevant information regarding miRNA recognition elements. Features characterizing the MRE were ranked according to discriminative and informative potential. Even though features concerning the miRNA-MRE duplex interaction confirmed to be discriminative, accessibility characteristics contribute to the identification of MREs according to their context inside the mRNA.

## Authors' contributions

AA, EF, EM participated in the design of the study and coordination. PR participated in the design of the study and she developed the algorithm. AA, EF, PR participated in writing the manuscript. All authors read and approved the final manuscript.
